# Work stress and competency among radiology residents: the mediating effect of resilience

**DOI:** 10.3389/fpubh.2024.1415351

**Published:** 2024-10-02

**Authors:** Lijun Shen, Yuanmei Lu, Yanrong He, Peicheng Wang, Yanhua Chen, Hange Li, Zhenghan Yang, Jingfeng Zhang, Zhenchang Wang, Maoqing Jiang, Jianjun Zheng, Jiming Zhu

**Affiliations:** ^1^Vanke School of Public Health, Tsinghua University, Beijing, China; ^2^School of Medicine, Tsinghua University, Beijing, China; ^3^National Centre for Health Professions Education Development, Graduate School of Education, Peking University, Beijing, China; ^4^Shengzhou High School, ZheJiang, China; ^5^Department of Radiology, Beijing Friendship Hospital, Capital Medical University, Beijing, China; ^6^Department of Radiology, Ningbo No. 2 Hospital, Ningbo, China; ^7^Institute for Healthy China, Tsinghua University, Beijing, China

**Keywords:** work stress, resilience, competency, radiology residents, mediating effect

## Abstract

**Background:**

Competency of health professionals stands as a fundamental element in ensuring the quality of care. Notably, work stress and resilience are found to be associated with competency of health professionals. However, the role of resilience between work stress and competency remains unexplored. This study aims to investigate competency, work stress and resilience of radiology residents, and to examine the mediating effect of resilience.

**Methods:**

A cross-sectional survey was conducted among 3,666 radiology residents from 31 provinces of China in 2021. The diagnostic radiology milestones were used to measure the competency. Results of work stress and resilience were derived from self-reports and assessment of the Connor Davidson Resilience Scale (CD-RISC) respectively. To examine the association between work stress, resilience, and competency, Spearman correlation analysis and hierarchical linear regression were employed. The mediating effect of resilience was tested by bootstrapping analysis.

**Results:**

Descriptive statistics show that the average score (mean ± SD) of work stress, competency and resilience among radiology residents were 1.55 ± 1.53 (range from 0 to 6), 28.14 ± 13 (range from 0 to 81) and 5.97 ± 1.92 (range from 0 to 8), respectively. Work stress was negatively associated with competency (*β* = −0.51, *p* < 0.001) and resilience (−0.57, *p* < 0.001). In particular, resilience mediated the relationship between work stress and competence, and the total mediating effect was −0.49 (= − 0.57 * 0.86), accounting for 49.06% of the total effect.

**Conclusion:**

Work stress is a significant contributor to competence among radiology residents. Resilience significantly mediated the association. This study highlights incorporating resilience training courses into the standardized residency training program to reduce intensive work stress and promote competency of radiology residents.

## 1 Introduction

Nowadays, high-quality healthcare is increasingly dependent on technological diagnostics (1). Radiologists are responsible for interpreting medical images, which directly influence patient diagnosis and treatment (2). In recent years, radiology has undergone increased case complexity and rapid technological advancements that place greater demands on radiologists. The additional administrative and documentation requirements tasks add more stress (3). Among radiology professionals, resident physicians constitute a significant portion (1). Medical residency is a crucial phase for physicians to transform towards autonomous practice with intense learning and increased responsibility (4). China embarked its nationwide standardized residency training (SRT) in 2013 (5). Though being pivotal in the development of proficient radiologists, SRT has been associated with various challenges, including high levels of work, learning pressure, work overload, and relatively low wages (6, 7). These issues underscore the importance of physical and psychological well-being of Chinese radiology residents.

Long-term exposure to work stress can reduce interest in work activities and initiatives, thereby negatively affecting performance (8). Previous studies showed that high work stress led to poor job performance (9–12). The stress experienced by dentists negatively influenced their diagnostic performance and potentially affected the quality of care and patient safety (11). Existing research has demonstrated that high levels of stress among radiology professionals not only severely impact their physical and mental health, leading to issues such as burnout, anxiety, depression, and musculoskeletal symptoms, but also reduce job satisfaction (12–15). However, there is a lack of research on the impact of stress on the job performance of radiology professionals. To assess job performance during residency training, the Accreditation Council for Graduate Medical Education (ACGME) has outlined six distinct competency domains along with corresponding milestones (16). Evaluating residents’ competency is essential to produce qualified physicians and to enhance overall healthcare quality and patient satisfaction (17). Therefore, it is necessary to study the impact of stress on resident competency, identify preventive factors that can mitigate the relationship between stress and competency and develop appropriate intervention measures.

Resilience is an important ability that mitigates the influence of stress (18). Resilience is defined as the positive psychological capacity to maintain good functioning after being exposed to stress, particularly as a strategy for thriving in the workplace (19). Previous studies indicated a significantly negative association between stress and resilience (20, 21), and a lack of resilience has been found to negatively affect the quality of clinical practice (22, 23). Accordingly, we hypothesized that resilience may mediate the association between work stress and competency. The stress-process model (SPM) can be employed as a robust explanation for the mediating effect (24). Specifically, the processes of perception, appraisal and coping would mitigate work stress, and bring positive or negative responses and outcomes (25, 26). As one of the coping strategies (27–29), resilience could mitigate the impacts of work stress on competence.

To our knowledge, few studies have examined the impact of stress on competence and the potential effect modifiers among radiology residents in China. Thus, based on a national survey among radiology residents, we aim to: (1) investigate the level of work stress, resilience and competency among radiology residents in China; (2) examine the associations between work stress, resilience and competency, and (3) explore the mediating role of resilience between work stress and competency. The study’s findings could provide valuable insights for the formulation of evidence-based policies aimed at mitigating work stress and enhancing the competency of radiology residents, thereby improving their health and productivity.

## 2 Materials and methods

### 2.1 Study design and participants

This cross-sectional study was part of a national survey of radiology residents in China, conducted by the Chinese Association of Radiologists (CAR) from December 1, 2020, to April 30, 2021. Questionnaires were distributed via a widely used online survey tool called “Wenjuanxing” in Chinese. Every radiology resident who participated in SRT during the period (*N* = 12,208) was invited, and 31% of them (*N* = 3,768) completed the online questionnaire (Participants were identified and recruited by the CAR). Four hundred and one out of 557 (72%) residency training sites across 215 cities of all 31 administrative regions in mainland China were included. To ensure the quality and representativeness of the results, we excluded 102 observations that had a completion time ≤ 300 s, missing values, and duplications. Observations that were out of the period of the study were also excluded. The final sample included 3,666 observations.

The anonymous online questionnaire took approximately 10–15 min to complete. An informed consent was obtained from each participant before they answered the questionnaire. This study was approved by the institution review board of Tsinghua University (No. 20210140).

### 2.2 Measures

All study variables were assessed in Chinese at the first measurement point.

#### 2.2.1 Work stress (independent variable)

Participants were asked to self-rate their frequency of unbearable pressure on a 7-point scale Likert scale (0 = “never” to 6 = “every day”). A higher score indicates a higher level of work stress.

#### 2.2.2 Resilience (potential mediator)

Resilience was assessed by the Connor Davidson Resilience Scale (CD-RISC) with two items based on the psychometric properties (30). The scale is a standardized and validated instrument (31, 32) that has been widely used in different populations. The two questions in the questionnaire are “I am able to adapt when changes occur,” and “I tend to bounce back after illness, injury, or other hardships.” A five-point Likert scale ranging from 0 (very disagree) to 4 (very agree) was utilized to evaluate these items, with a total score ranging from 0 to 8 (0 indicates the lowest resilience level).

#### 2.2.3 Competency (dependent variable)

We used diagnostic radiology milestones to measure competency (33), which provides a structured framework that tracks the development of a radiologist’s skills and knowledge attainment over time. Many accreditation bodies and certification boards, such as the ACGME used milestones as a benchmark for evaluating residency programs. Meeting these milestones is often necessary for board certification and practice. Specifically, the diagnostic radiology milestones include six dimensions of competency in 24 sub-competencies: patient care (PC), medical knowledge (MK), systems-based practice (SBP), practice-based learning and improvement (PBLI), interpersonal and communication skills (ICS) and professionalism. Based on the discussions and recommendations of the experts from CAR, 9 out of the 24 sub-competencies have been selected for our research, considering the current development of residency training in China. The Cronbach alpha reliability coefficients (*α* = 0.93) for 9 sub-competencies indicate the reliability of our sample for further statistical analysis. According to the diagnostic radiology milestones (16), a 10-point Likert scale (0–9 scores) was used to evaluate the 9 sub-competencies, with total scores ranging from 0 to 81. A higher the score indicates a higher level of competency.

#### 2.2.4 Demographic variables

Demographic factors include gender (male and female), age (year), region (East, Middle, West, and Northeast), major (Clinical medicine, Medical imaging, Others), residency training site (General tertiary A, Specialist tertiary A, General tertiary B, Others), residency year (the first year, the second year, and the third year), education level (Bachelor’s degree, Master’s degree, and Doctoral degree) and marital status (Married, Unmarried, and Others).

### 2.3 Data analysis

This section describes the statistics of the sample, as well as the statistical approach used for the main analysis. In particular, analysis of variance (ANOVA) was used to compare work stress, resilience and competency against demographic variables. Considering the sample distribution, Spearman correlation analysis was used to determine the correlations between work stress, resilience and the six domains of competency among radiology residents. The hierarchical linear regression with hospital fixed effects was used to investigate the associations between work stress, resilience and competency controlling for demographic variables, and the Enter method was adopted. Model 1 was established with competency as the dependent variable and work stress as the independent variable. Model 2 was similarly established with resilience as the dependent variable and work stress as the independent variable. Model 3 included competency as the dependent variable, with both work stress and resilience as independent variables. Standard errors (SEs) were clustered at the hospital level to adjust for possible autocorrelations between participants’ working in the same place. The three-step procedure proposed by Baron and Kenny was used to evaluate the mediating effect of resilience (34). The mediating effects exist when independent variable (work stress) significantly predicts dependent variable (competency) and mediator (resilience), and mediator (resilience) significantly predicts dependent variable (competency) while controlling for independent variable (work stress).

In addition, the bootstrapping procedure defined by Preacher and Hayes (35) was used to further test the mediating effect and 95% confidence intervals (CI) were estimated. The assessment was sampled 1,000 times, and control variables such as age, gender and major were introduced in the model as covariates. The indirect effect was considered statistically significant if the 95% CI did not contain zero (36). The significance level was set at the 0.05 level (two-tailed). All analyses were performed with Stata, version 16.1.

## 3 Results

### 3.1 Background characteristics

Table 1 summarized the demographic characteristics of the participants. The mean age of the residents was 27.3 years old. The older the residents were, the higher level of resilience (*p* = 0.009) and competency (*p* < 0.001) they had. Out of the total of 3,666 radiology residents, 41% originated from the eastern region, which was identified to have the highest level of work stress (*p* = 0.02). Almost all of the residents were trained in tertiary hospitals (97%) and had a bachelor’s degree (92%). The majority of participants were unmarried (77%), and this group had the lowest score of competency (*p* < 0.001). Males reported experiencing more work stress than females (*p* < 0.001), but they also exhibited higher levels of resilience and competency (*p* < 0.001). A majority of the residents (85%) specialized in medical imaging, and they exhibited a higher competency than counterparts in clinical medicine and other disciplines (*p* < 0.001). Residents in their second year of training experienced the highest level of work stress and the lowest level of resilience (*p* < 0.001). In addition, the score of competency increased along with the progress of training (*p* < 0.001).

**Table 1:** Demographic, work stress, resilience and competency variables of the participants (*N* = 3,666).

	*N* (%)	Work stress (*M* ± SD)	*p*-value	Resilience (*M* ± SD)	*p*-value	Competency (*M* ± SD)	*p*-value
**Region**			0.023		0.016		0.405
Northeast	218 (5.95)	1.55 ± 1.73		6.28 ± 1.81		28.64 ± 14.20	
East	1,485 (40.51)	1.62 ± 1.54		5.95 ± 1.97		27.70 ± 12.68	
Middle	743 (20.27)	1.57 ± 1.55		5.85 ± 1.89		28.42 ± 13.43	
West	1,220 (33.28)	1.44 ± 1.45		6.03 ± 1.88		28.42 ± 13.22	
**Gender**			<0.001		0.392		<0.001
Male	1,539 (41.98)	1.69 ± 1.65		6.01 ± 1.97		29.39 ± 13.68	
Female	2,127 (58.02)	1.44 ± 1.43		5.95 ± 1.88		27.23 ± 12.6	
**Major**			0.212		0.437		<0.001
Clinical medicine	528 (14.4)	1.63 ± 1.62		5.94 ± 1.98		25.58 ± 13.51	
Medical imaging	3,102 (84.62)	1.54 ± 1.51		5.97 ± 1.91		28.58 ± 12.98	
Others	36 (0.98)	1.25 ± 1.42		6.36 ± 1.79		27.81 ± 13.72	
**Residency training site**			0.590		0.786		0.762
General tertiary A	3,550 (96.84)	1.55 ± 1.53		5.97 ± 1.92		28.16 ± 13.08	
Specialist tertiary A	80 (2.18)	1.32 ± 1.39		6.15 ± 1.76		27.54 ± 13.83	
General tertiary B	27 (0.74)	1.56 ± 1.48		6.11 ± 2.36		26.37 ± 12.75	
Others	9 (0.25)	1.78 ± 1.99		5.67 ± 2.83		31.22 ± 16.78	
**Residency year**			0.006		<0.001		<0.001
The 1st year	1,285 (35.05)	1.45 ± 1.48		6.15 ± 1.85		24.10 ± 11.84	
The 2nd year	1,179 (32.16)	1.65 ± 1.59		5.76 ± 1.99		27.97 ± 12.4	
The 3rd year	1,202 (32.79)	1.55 ± 1.52		6.00 ± 1.9		32.61 ± 13.63	
**Age, *M* (SD)**	27.3 (2.58)		0.789		0.009		<0.001
<=26	1,625 (44.33)	1.54 ± 1.53		6.06 ± 1.91		25.60 ± 12.17	
26–29	1,390 (37.92)	1.54 ± 1.52		5.96 ± 1.92		29.41 ± 12.93	
>29	651 (17.76)	1.58 ± 1.54		5.78 ± 1.93		31.75 ± 14.42	
**Educational level**			0.094		0.057		<0.001
Bachelor’s degree	3,375 (92.06)	1.56 ± 1.54		5.99 ± 1.92		27.90 ± 12.96	
Master’s degree	229 (6.25)	1.34 ± 1.39		5.86 ± 1.92		30.56 ± 14.27	
Doctoral degree	62 (1.69)	1.69 ± 1.62		5.45 ± 2.02		32.13 ± 15.11	
**Marital status**			0.428		0.872		<0.001
Married	820 (22.37)	1.49 ± 1.54		5.94 ± 1.92		30.79 ± 14.13	
Unmarried	2,829 (77.17)	1.56 ± 1.53		5.98 ± 1.92		27.35 ± 12.68	
Others	17 (0.46)	1.59 ± 1.58		6.06 ± 1.89		30.88 ± 14.21	

### 3.2 Spearman correlations between work stress, resilience and competency

Table 2 provided detailed information on the correlation analysis. Resilience was negatively associated with work stress (*r* = −0.48, *p* < 0.001), while positively related to competency (*r* = 0.13, *p* < 0.001). The bivariate correlation analysis showed a negative correlation between work stress and competency (*r* = −0.10, *p* < 0.001). The six dimensions of competency had significantly negative associations with work stress and positive associations with resilience (*p* < 0.001). Notably, interpersonal and communication skills had the strongest correlation with work stress and resilience (*r* = −0.12, *p* < 0.001; *r* = 0.16, *p* < 0.001).

**Table 2:** Spearman correlations between work stress, resilience and competency.

Dimensions		*M* (SD)	Range	1	2	3	4	5	6	7	8	9
1	Work stress	1.55 (1.53)	0–6	1								
2	Resilience	5.97 (1.92)	0–8	−0.48***	1							
3	TC^a^	28.14 (13.10)	0–81	−0.10***	0.13***	1						
4	PC^b^	5.69 (2.90)	2–18	−0.05**	0.07***	0.81***	1					
5	MK^c^	6.73 (3.25)	2–18	−0.09***	0.11***	0.87***	0.69***	1				
6	SBP^d^	5.92 (3.37)	2–18	−0.09***	0.12***	0.90***	0.66***	0.74***	1			
7	PBLI^e^	3.01 (1.79)	1–9	−0.08***	0.10***	0.85***	0.61***	0.67***	0.77***	1		
8	Pro^f^	3.28 (1.87)	1–9	−0.12***	0.15***	0.85***	0.59***	0.67***	0.73***	0.75***	1	
9	ICS^g^	3.51 (2.07)	1–9	−0.12***	0.16***	0.83***	0.55***	0.64***	0.68***	0.70***	0.77***	1

a Total Competency (TC) is the sum of the six dimensions of competency.

b Patient Care (PC).

c Medical Knowledge (MK).

d Systems-Based Practice (SBP).

e Practice-Based Learning and Improvement (PBLI).

f Professionalism (Pro).

g Interpersonal and Communication Skills (ICS).

*p < 0.05; **p < 0.01; ***p < 0.001.

### 3.3 Mediation effects of resilience

**Figure 1:**
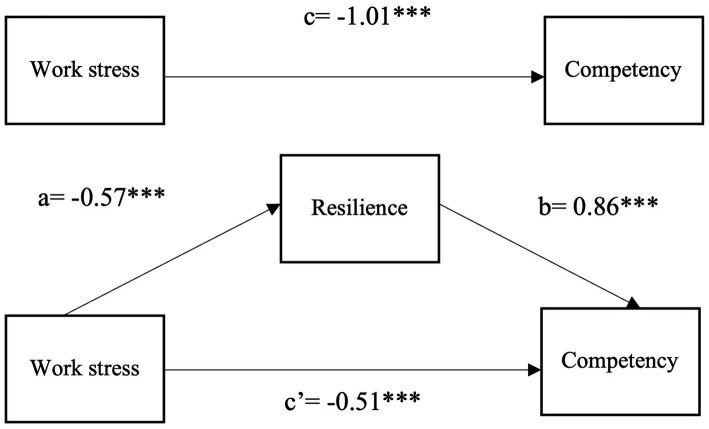
The mediation models among work stress, resilience and competency.

Table 3 and Figure 1 present the results of hierarchical linear regressions. In model 1, we found a significantly negative association between work stress and competency (*β* = −1.01, *p* < 0.001). Results in Model 2 showed that the greater the stress is, the lower level of resilience would be (*β* = −0.57, *p* < 0.001). Model 3 examines the mediating effects of resilience. The association between work stress and competency remained significantly negative (*β* = −0.51, *p* < 0.001) after being mediated by resilience. The total mediating effect between work stress and competency was −0.494 (*β* = −0.57 * 0.86), accounting for 49.06% of the total effect.

**Table 3:** The mediating effect of resilience between work stress and competency.

Variable	Model 1 (Y = Competency)	Model 2 (Y = Resilience)	Model 3 (Y = Competency)
	*β* (SE)	*β* (SE)	*β* (SE)
Resilience	NA	NA	0.86***
			**(0.13)**
Work stress	−1.01***	−0.57***	−0.51***
	**(0.15)**	**(0.03)**	**(0.16)**
**Region (ref = Northeast)**			
East	7.41***	−1.17***	8.42***
	(0.40)	(0.10)	(0.44)
Middle	4.15***	−0.76***	4.80***
	(0.52)	(0.03)	(0.53)
West	8.46***	−0.77***	9.12***
	(0.52)	(0.07)	(0.47)
**Gender (ref = Male)**			
Female	−2.07***	−0.21***	−1.89***
	(0.49)	(0.07)	(0.48)
**Major (ref = Clinical medicine)**			
Medical imaging	3.67***	−0.09	3.75***
	(0.76)	(0.08)	(0.76)
Others	3.80	0.08	3.73
	(2.95)	(0.41)	(2.81)
**Residency training site (ref = General tertiary A)**			
Specialist tertiary A	−1.40	0.26	−1.62
	(2.00)	(0.32)	(2.07)
General tertiary B	−4.15	0.27	−4.38
	(4.36)	(0.99)	(3.56)
Others	6.11	0.27	5.88
	(8.25)	(1.90)	(6.62)
**Residency year (ref = The 1st year)**			
The 2nd year	3.28***	−0.23***	3.48***
	(0.57)	(0.08)	(0.57)
The 3rd year	7.77***	−0.05	7.82***
	(0.65)	(0.08)	(0.64)
Age	0.55***	−0.01	0.56***
	(0.14)	(0.02)	(0.14)
**Education (ref = Bachelor’s degree)**			
Master’s degree	2.00*	−0.18	2.15**
	(1.04)	(0.15)	(1.03)
Doctoral degree	5.99**	−0.24	6.20**
	(2.54)	(0.24)	(2.50)
**Marital status (ref = Married)**			
Unmarried	−0.55	0.08	−0.62
	(0.67)	(0.09)	(0.67)
Others	1.71	0.11	1.61
	(4.27)	(0.46)	(4.35)
Cons	2.73	8.15***	−4.31
	(4.27)	(0.47)	(4.20)

All models were controlled for the demographic variables; The R-squared of Model 1, Model 2 and Model 3 were 0.24, 0.34, and 0.25, respectively; Robust standard error in parentheses; *p < 0.05; **p < 0.01; ***p < 0.001. The bold values represent the statistical values of the mediation effect analysis, which are included in the discussion.

The bootstrap tests additionally supported that resilience mediated the relationship between work stress and competency. The direct effect illustrated by Table 4 indicated a negative influence of work stress on competency scores [coefficient = −0.48, 95% CI = (−0.79, −0.16)]. Additionally, after adding the resilience, the direct effect remains strong [coefficient = −0.51, 95% CI = (−0.66, −0.36)].

**Table 4:** Direct and indirect effect analysis (bootstrap estimation).

Pathway	Coefficient	SE	95% CI	
			Lower	Upper
**Direct effects**				
Work stress—Competency	−0.48	0.16	−0.79	−0.16
**Indirect effects**				
Work stress—Resilience—Competency	−0.51	0.08	−0.66	−0.36

## 4 Discussion

This study first investigated the mediating role of resilience in the relationship between work stress and competency among radiology residents. It provides empirical evidence based on first-hand and individual-level data collected from 401 hospitals participating in the SRT program. Firstly, same as existing literature, we find that work stress was negatively associated with competency. Secondly, we show that resilience mediated the negative association between work stress and competency. Specifically, this paper finds that resilience exhibited a negative correlation with work stress, while demonstrating a positive connection with competency. The findings highlight the potential significance of resilience as a pivotal factor in mitigating work-related stress and enhancing the level of competency among radiology residents. Consequently, the integration of resilience training into the residency program becomes imperative to optimize the advantageous outcomes associated with resilience.

The mean score of work stress was 1.55 (range: 0–6) in our study, meaning that Chinese radiologists experience insufferable stress almost once a month on average. Such a level of perceived stress is relatively low compared to other departments in China (37). One possible explanation is that radiology is not a clinical department in China, but rather a medical technical support department in a relatively low work stress level with shorter working hours (38, 39). For instance, surgical residents worked 84.3 h per week, which is much longer than 52.2 h of radiology residents (40). Additionally, the 12-h on-call shifts for radiology residents are not as strict as the 24-h on-call shifts for residents in other specialties such as internal medicine (41). The overall mean score of resilience was 5.97 (range: 0–8) in radiology residents, which is relatively higher than those from other disciplines in China (25) but lower than the international level of resilience (30, 42). It was found that resilience could attribute to the high personal accomplishments and job satisfaction among professional physicians (43, 44), as well as to the help of resilience training programs (45, 46).

Our study found that the overall mean score of competency was 28.14 (range: 0–81). The mean value for sub-competencies was 3.13, which corresponds to level 2 in the ACGME standards. Level 4 is considered by the ACGME to be a standard for training qualification (44), and therefore a lower level of competency in our study. The results were also lower than those of residents from other specialties, such as family medicine and internal medicine (47–49). One possible reason is the strict residency training requirements in countries such as the U.S. where radiology residents must complete 5 years of training and undergo periodic testing (50, 51). Furthermore, consistent with our previous study, inadequate training on competency such as interpersonal and communication skills (35) may contribute to low competency (25, 49). Moreover, radiology is often viewed as an adjunct to quality care within the healthcare system, with limited involvement in direct patient treatment. This lack of face-to-face interactions with clinical colleagues can contribute to suboptimal radiology practice (2, 52, 53). To keep up with the worldwide pace, the current residency training of radiology calls for instructional reforms to prioritize competency-driven approaches to instructional design.

There was a negative association between work stress and competency. Residents who have much work stress may find it difficult to maintain a balanced and healthy psychological state and fully devote themselves to their work, leading to insufficient competency (54, 55). Specifically, prolonged exposure to high work stress can lead to mental health issues like depression and burnout, significantly impairing doctors’ ability to perform effectively and reducing the quality of patient care (56–58). Moreover, work pressure can also cause physiological issues such as insomnia and musculoskeletal symptoms, further weakening doctors’ physical and mental strength to maintain optimal performance (15, 59). Although the negative impact of work stress has been discussed for several years, mandating limits on work hours may not be more effective than encouraging radiologists to self-regulate. Therefore, it is crucial to understand how radiology residents can leverage their personal resources to prevent the negative impacts of stress.

According to our mediation effect analysis, we observed a negative association between work stress and resilience (*β* = −0.61; *p* < 0.001). This connection could potentially be linked to the sensitization mechanism of resilience (60). Those who become more sensitive to stress might be more prone to additional stress or might experience a reduction in their ability to resist stress (61–63). The ability for resilience might be diminished by exposure to multiple or persistent stressors (64). In addition, consistent with prior findings (65, 66), we find that there was a positive association between resilience and competency (*β* = 0.83; *p* < 0.001). Individuals with better resilience exhibit more positive emotions (67) and greater satisfaction and engagement (20), which contribute to a higher level of competency and better professional performance. After the addition of resilience, the association between work stress and competency was still significantly negative (*β* = −0.46, *p* < 0.01), and the mediating effect accounts for 49.06%. This result demonstrated that work stress not only directly predicts competency, but also indirectly affects competency through resilience.

Resilience could be a protective factor to alleviate the negative impacts of stress (68–72) through promoting life satisfaction and well-being, improving sleep quality, and enhancing work performance. Moreover, to examine the robustness of the result, we conduct analyses by focusing on the association between stress and six dimensions of competency (Supplementary Tables S1–S6). In particular, we showed that the effect of work stress on patient care was fully mediated by resilience. For the other five dimensions, the proportion of mediating effects ranged from 40 to 55%. Based on these results, it would be necessary to involve residents in resilience training programs, such as CBT (cognitive behavioral therapy), mindfulness-based training, and a combination of CBT and mindfulness training (73, 74).

There are some limitations in this study. The model might be susceptible to potential omitted variable bias if there are unobserved personal characteristics that simultaneously impact both work stress and competency. However, we have taken steps to mitigate this by incorporating numerous characteristics at the individual’s level, hospital’s level, and regional level, aiming to minimize the influence of unobserved confounding factors. Another limitation pertains to the potential issue of reversed causality. If a portion of competency is determined by exogenous factors, such as genetic influences, it’s conceivable that radiology residents with lower competency levels could experience heightened stress in their daily work. Consequently, relying solely on professional resilience training might not fully address the underlying work stress issue. In the future, cohort studies or panel data (i.e., with consideration of individual fixed effects to capture any potential time-invariant confounders) could be used for in-depth causal inference analysis.

In conclusion, it is alarming that radiology residents in China have reported a lower level of competency. Work stress is an important predictor of residents’ competency, while resilience has significant mediating effects on competency. Our findings suggest that though it is important to redcuce work stress, policy makers and hospital administrators need to be aware of the mediating role of resilience on radiology residents. It is essential to integrate resilience training into the curriculum of SRT programs, which may be helpful and cost-effective to help improve competency of residents and enhance their adaptability to the intense work stress from the healthcare settings.

## Data Availability

The original contributions of this study are provided in the article and supplementary materials. Further inquiries can be directed to the corresponding author.
