# Supplementary material for: Work stress and competency among radiology residents: the mediating effect of resilience
Source: Front Public Health. 2024 Oct 2;12:1415351. doi: 10.3389/fpubh.2024.1415351 (PMC11479909; doi:10.3389/fpubh.2024.1415351)
Supplement: Supplementary file 1 [file Table_1.docx]

**Table S1. The mediating effect of resilience between work stress and Patient Care of Competency**

| Variable | Model 1  (Y=Competency) | Model 2  (Y= Resilience) | Model 3  (Y=Competency) |
| --- | --- | --- | --- |
|  | β (SE) | β (SE) | β (SE) |
| Resilience | NA | NA | 0.12*** |
|  |  |  | (0.03) |
| Work stress | -0.11** | -0.57*** | -0.04 |
|  | (0.03) | (0.03) | (0.04) |
| Cons | 0.24 | 8.15*** | -0.73 |
|  | (0.94) | (0.47) | (0.96) |

Note. After the addition of resilience, the link from work stress to competency has become insignificant (β= -0.04, p =.230), which preliminarily demonstrated that resilience played a **complete mediating role** between work stress and Patient Care of Competency. All models adjusted for region, gender, major, residency training site tier, residency training year, age, education and marital status; The R-squared of Model 1, Model 2 and Model 3 were 0.24, 0.34 and 0.24, respectively; *p<.05; **p<.01; ***p<.001.

**Table S2. The mediating effect of resilience between work stress and medical knowledge of Competency**

| Variable | Model 1  (Y=Competency) | Model 2  (Y= Resilience) | Model 3  (Y=Competency) |
| --- | --- | --- | --- |
|  | β (SE) | β (SE) | β (SE) |
| Resilience | NA | NA | 0.19*** |
|  |  |  | (0.03) |
| Work stress | -0.22*** | -0.57*** | -0.12** |
|  | (0.04) | (0.03) | (0.04) |
| Cons | 1.15 | 8.15*** | -0.36 |
|  | (1.04) | (0.47) | (1.05) |

Note: After the addition of resilience, the link from work stress to competency was still significant (β = -0.12, p = .007), which preliminarily demonstrated that resilience played a mediating role between work stress and medical knowledge of competency. The mediating effect of work stress on resident competency through resilience was -0.11 (=0.19 *-0.57), accounting for 47.97% of the total effect; All models adjusted for region, gender, major, residency training site tier, residency training year, age, education and marital status; The R-squared of Model 1, Model 2 and Model 3 were 0.23, 0.34 and 0.24, respectively; *p<.05; **p<.01; ***p<.001.

**Table S3. The mediating effect of resilience between work stress and systems-based practice of Competency**

| Variable | Model 1  (Y=Competency) | Model 2  (Y= Resilience) | Model 3  (Y=Competency) |
| --- | --- | --- | --- |
|  | β (SE) | β (SE) | β (SE) |
| Resilience | NA | NA | 0.20*** |
|  |  |  | (0.04) |
| Work stress | -0.21*** | -0.57*** | -0.09* |
|  | (0.04) | (0.03) | (0.04) |
| Cons | -0.65 | 8.15*** | -2.27* |
|  | (1.15) | (0.47) | (1.11) |

Note: After the addition of resilience, the link from work stress to competency was still significant (β = -0.09, p = .031), which preliminarily demonstrated that resilience played a mediating role between work stress and systems-based practice of competency. The mediating effect of work stress on resident competency through resilience was -0.12 (=0.20 *-0.573), accounting for 55.63% of the total effect; All models adjusted for region, gender, major, residency training site tier, residency training year, age, education and marital status; The R-squared of Model 1, Model 2 and Model 3 were 0.21, 0.34 and 0.22, respectively; *p<.05; **p<.01; ***p<.001.

**Table S4. The mediating effect of resilience between work stress and Practice-Based Learning and Improvement of Competency**

| Variable | Model 1  (Y=Competency) | Model 2  (Y= Resilience) | Model 3  (Y=Competency) |
| --- | --- | --- | --- |
|  | β (SE) | β (SE) | β (SE) |
| Resilience | NA | NA | 0.08*** |
|  |  |  | (0.02) |
| Work stress | -0.11*** | -0.57*** | -0.07** |
|  | (0.02) | (0.03) | (0.02) |
| Cons | 0.21 | 8.15*** | -0.46 |
|  | (0.56) | (0.47) | (0.57) |

Note: After the addition of resilience, the link from work stress to competency was still significant (β = -0.07, p = .007), which preliminarily demonstrated that resilience played a mediating role between work stress and Practice-Based Learning and Improvement of competency. The mediating effect of work stress on resident competency through resilience was -0.047 (=0.08 *-0.573), accounting for 41.22% of the total effect; All models adjusted for region, gender, major, residency training site tier, residency training year, age, education and marital status; The R-squared of Model 1, Model 2 and Model 3 were 0.19, 0.34 and 0.20, respectively; *p<.05; **p<.01; ***p<.001.

**Table S5. The mediating effect of resilience between work stress and Professionalism of Competency**

| Variable | Model 1  (Y=Competency) | Model 2  (Y= Resilience) | Model 3  (Y=Competency) |
| --- | --- | --- | --- |
|  | β (SE) | β (SE) | β (SE) |
| Resilience | NA | NA | 0.13*** |
|  |  |  | (0.02) |
| Work stress | -0.15*** | -0.57*** | -0.08** |
|  | (0.02) | (0.03) | (0.02) |
| Cons | 0.70 | 8.15*** | -0.34 |
|  | (0.63) | (0.47) | (0.61) |

Note: After the addition of resilience, the link from work stress to competency was still significant (β = -0.08, p = .001), which preliminarily demonstrated that resilience played a mediating role between work stress and Professionalism of competency. The mediating effect of work stress on resident competency through resilience was -0.07 (=0.13 *-0.57), accounting for 47.25% of the total effect; All models adjusted for region, gender, major, residency training site tier, residency training year, age, education and marital status; The R-squared of Model 1, Model 2 and Model 3 were 0.19, 0.34 and 0.20, respectively; *p<.05; **p<.01; ***p<.001.

**Table S6. The mediating effect of resilience between work stress and Interpersonal and Communication Skills of Competency**

| Variable | Model 1  (Y=Competency) | Model 2  (Y= Resilience) | Model 3  (Y=Competency) |
| --- | --- | --- | --- |
|  | β (SE) | β (SE) | β (SE) |
| Resilience | NA | NA | 0.15*** |
|  |  |  | (0.02) |
| Work stress | -0.20*** | -0.57*** | -0.12*** |
|  | (0.03) | (0.03) | (0.03) |
| Cons | 1.07 | 8.15*** | -0.14 |
|  | (0.66) | (0.47) | (0.69) |

Note: After the addition of resilience, the link from work stress to competency was still significant (β = -0.12, p < .001), which preliminarily demonstrated that resilience played a mediating role between work stress and Interpersonal and Communication Skills of competency. The mediating effect of work stress on resident competency through resilience was -0.09 (=0.15 *-0.57), accounting for 42.27% of the total effect; All models adjusted for region, gender, major, residency training site tier, residency training year, age, education and marital status; The R-squared of Model 1, Model 2 and Model 3 were 0.18, 0.34 and 0.19, respectively; *p<.05; **p<.01; ***p<.001.
